# High Risk Situations Predicting Relapse in Self-Referred Addicts to Bushehr Province Substance Abuse Treatment Centers

**DOI:** 10.5812/ijhrba.16381

**Published:** 2014-06-15

**Authors:** Ebrahim Shafiei, Agha Fatemeh Hoseini, Alireza Bibak, Mohammad Azmal

**Affiliations:** 1 Department of Cardiac Surgery, Bushehr University of Medical Sciences, Bushehr, IR Iran; 2 Department of Biostatistics, School of Public Health, Iran University of Medical Sciences, Tehran, IR Iran; 3 Department of Psychology, Bushehr University of Medical Sciences, Bushehr, IR Iran; 4 Department of Mental Health, Bushehr University of Medical Sciences, Bushehr, IR Iran

**Keywords:** Recurrence, Substance Abuse Treatment Centers, High risk situations

## Abstract

**Background:**

Relapse prevention is a medical intervention designed for educating cognitive and behavioral skills to avoid continued drug abuse and relapse.

**Objectives:**

This study examined high risk situations for relapse for self-referred addicts are related in Bushehr province substance abuse treatment centers.

**Patients and Methods:**

The present study is descriptive cross-sectional. The sample size consisted of 609 self-referred addicts to Bushehr province substance abuse centers. IDTS Marlatt questionnaire was used. Analytical and descriptive statistics were used to analyze the data.

**Results:**

The findings showed that 73.1% of addicts have used substance during the past 12 months, and 72% have experienced a full relapse. Unpleasant emotions and physical discomfort was the most important reason for relapse and testing personal control and pleasure emotions the least important reason. Interpersonal factors have also a great role in this regard.

**Conclusions:**

Considering the high rates of relapse, more attention should be paid to reasons for relapse. It seems necessary that both clinical and psychological approaches would be undertaken simultaneously.

## 1. Background

Addiction is a psychological and behavioral syndrome which is associated with a strong desire to use drugs (1). Some people who use drugs and have entered to addiction’s process are attempting to quit or control drug abuse, when they become aware of its negative side effects. But a small number of them who have stopped their drug abuse, can not fully observe their abstinence and will relapse again (2). Addiction is a multi dimensional disease and is often associated with many problems such as frequent relapse (3). Drug abuse is known as a chronic, long term relapsing process. Unfortunately, little attention has been paid to this issue up to now (4).

Marlatt and Gordon have estimated the likelihood of return to drug abuse to be about 50 percent by the most optimistic estimation. According to them, this probability varies from 60 to 90 % (5). In Iran, fifty percent of recovered addicts have a tendency to resume drug abuse (6). The relapse concept has changed over the years and it is now as a failure in person’s recovery. As Dennis et al have noted, relapse is the result of previous unhealthy actions that predisposes the person to relapse. Normally, people who relapse show signs of change in their attitudes, thoughts, feelings and actions (7). In a study conducted in Iran, 80% of addicts relapsed at the first 6 months (8).

Several models have been proposed for relapse, but the most comprehensive is the cognitive behavioral approach (4, 5). In this regard, the concept of “high risk situations” has emerged and two main categories of high-risk situations were identified: intrapersonal factors including negative emotions (NE) , physical discomfort (PD) , urge and temptation (UT), positive emotions (PE), test of personal control (TPC) and, interpersonal factors, including conflict with others (CO), social pressure (SP), pressure times (PT) (5). Relapse prevention is a medical intervention designed for educating cognitive and behavioral skills to avoid relapse. Marllat's relapse prevention (RP) model was developed after over twenty years of his research and is updated continually based on empirical findings (9). Although other relapse models exist, valid results have been achieved using this model in well-designed and well- controlled studies. This model is based on a number of high-risk situations that can make people aware of the potential for relapse. The high-risk situations include people, places, feelings and thoughts which can lead to relapse. In addition, there are numerous factors aside from these high-risk situations such as destructive thinking patterns, unbalanced lifestyle and lack of planning. These combined factors undermines individuals’ decisions even before encountering the situation (5). The more risk factors a person has, the higher is the likelihood of drug abuse (10). The RP model includes specific intervention strategies which allow the therapist and the client to examine each step of the relapse process. This intervention includes identifying specific high-risk situations and coping skills to deal with the situation, increasing self efficacy, handling relapse, and reconstructing clients’ perceptions of the relapse process and therefore reducing the risk of relapse (6).

In recent years, the relapse prevention model is widely used throughout the world. Marlatt and Gordon in a study about highest risk situations for relapse among the youth indicated that interpersonal and intrapersonal factors compromised 58% and 42% of the original sample, respectively. Unpleasant emotions were the most frequently reported high risk situation (37%) (5). El, Sheikh investigated some relapse related situations in alcoholics and heroin addicts. They found that physical discomfort, social pressure and pleasant times with others situations, had the high mean scores in both groups (9). In Hartwell’s study performed among opiate addicts, the highest mean scores were related to physical discomfort, conflicts with others and testing the personal control (11). In another study from Iran, the most prevalent reason of relapse was unpleasant emotions and spending pleasant times with others (12). Despite extensive studies in our country, we could not find any study that had extensively evaluated high- risk situations for relapse.

## 2. Objectives

The goal of this study was to explore relapse high-risk situations among self-referred addicts to the Bushehr province substance abuse treatment centers in the south of Iran.

## 3. Patients and Methods

A cross-sectional descriptive study was conducted in 2012 to assess high risk situations for relapse in self-referred addicts in Bushehr province substance abuse treatment centers in Iran. The study population consisted of self-referred addicts that have been treated in public and private substance abuse treatment centers. In total, 609 self- referred addicts participated in this study (response rate = 65%).

A self expression questionnaire was used to collect the data. This instrument is consisted of two parts. The first part includes the demographic variables such as age, sex, marital status and relevant information including the history of imprisonment and history and frequency of relapses. The second part of the inventory of drug-taking situations (IDTS), a self report questionnaire that investigates the profile of drug-taking situations over the past year in eight high risk situations, was used (13). The IDTS was developed by Annis and Martin (13) and some researcher have used it in their work. In Iran, this tools was used to identify individual and social factors associated with relapse in 2004 (12). A five point Likert scale, ranging from Never (1), rarely (2), little (3), often (4) and always (5) was administered to gauge high risk situations and for drawing addicts’ profile, this scale was scored by percent (never-0%, rarely-25%, little-50%, often-75% and always-100%) based on the current literature. Validity is established using expert’s opinions and Instruments’ Reliability was measured by test-retest. For this reason, questionnaires were completed by 30 people of the same sample batch and after two weeks repeated; the resulting correlation coefficient was calculated to be about of 0.86.

 The collected data were analyzed using SPSS software (Version 17). Descriptive statistic indicators (frequency, mean, variance, standard deviation and etc.) were used in the analysis of data. The significance levels were set at P < 0.05.

## 4. Results

Six hundred and nine self-referred addicts to Bushehr province substance abuse treatment centers ranging in age from 16 to 70 (Mean ± SD = 34.5 ±9.81) participated in the study. The highest frequency age groups were aged 21-30 and the lowest were aged ≥ 60 years. Most participants in this study were male (97.8%). Regarding the level of education, most participants had a secondary school education. Of the participants, 81.3% were employed; 60.2% of people were single, 35.3% married and 4.5% separated, respectively; 32.7% of individuals mentioned a history of imprisonment. The primary drug of choice was opiate (52.4%).

**Figure 1.**
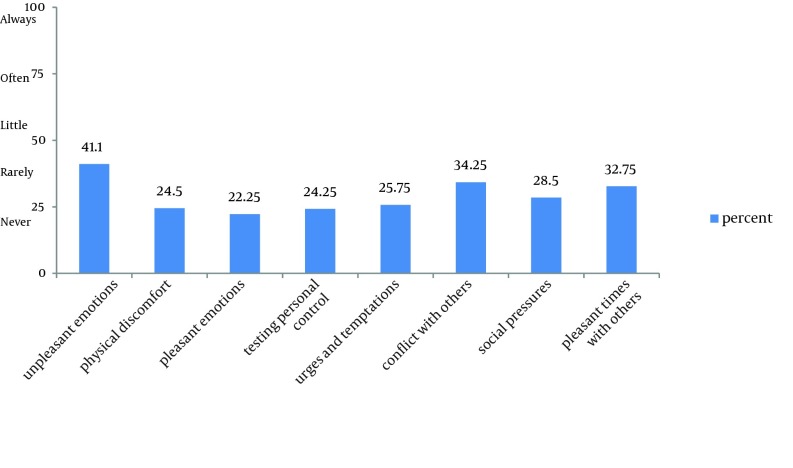
Frequency of High Risk Relapse situations by Percentage

From the under treatment addicts, 73.1% reported that they had abused drugs over the past 12 months. The mean score of substance consumption was 3.11 out of 4 in the same period. Overall, 72% of subjects had a history of relapse and 27.1% of them had experienced relapse once, 30.9% twice and the others more two times. In terms of treatment, 84.8% noted a history of detoxification, and 65.3% MMT (methadone maintenance therapy) as well. However, considering the type of treatment after relapse 38.5% of them received medication, 14% psychological therapy, and 47.5% had received both types of treatment. According to Table 1, 50.2% of individuals’ response to questions about these was ‘never’. A pleasant emotion (with frequency of 61.1%) was the greatest high risk situation. The highest and least mean scores associated with high risk situations were related to pleasant and unpleasant emotions, respectively. Figure 1 shows the overall profile of high risk situations. By comparing this profile with a standard profile, we found that is similar to an ordinary profile and addicts have little confidence (less than 50%), when facing the high risk situations. In this regard, unpleasant emotions and physical discomfort were the most important situations and testing personal control and pleasant emotions were the least important situations predisposing to relapse. According to our findings, the mean of intrapersonal factors (NE, PD, UT, PE and TPC situations) and interpersonal factors (CO, SP and PT situations) were calculated to be 1.18 and 1.28, respectively (from maximum 4).

**Table 1.** Inventory of Drug-Taking Situations (IDTS) Items Results ^a^

High-Risk Situations	Never	Rarely	Little	Often	Always	Results
**Unpleasant emotions**	220 (37.7)	111 (19)	35 (6)	89 (15.3)	128 (22)	1.61 ± 1.64
**Physical discomfort**	255 (43.7)	116 (19.9)	42 (7.2)	73 (12.5)	97 (16.6)	1.54 ± 1.38
**Pleasant emotions**	356 (61.1)	87 (14.9)	33 (5.7)	62 (10.6)	45 (7.7)	1.33 ± .89
**Testing personal control**	337 (57.6)	92 (15.7)	43 (7.4)	58 (9.9)	55 (9.4)	1.37 ± .97
**Urges and temptations**	333 (57.2)	72 (12.4)	59 (10.1)	57 (9.8)	61 (10.5)	1.42 ± 1.03
**Conflict with others**	258 (44.4)	100 (17.2)	56 (9.6)	81 (13.9)	86 (14.8)	1.51 ± 1.37
**Social pressures**	305 (52.7)	95 (16.4)	47 (8.1)	53 (9.2)	79 (13.6)	1.48 ± 1.14
**Pleasant times with others**	276 (47.7)	99 (17.1)	49 (8.6)	54 (9.3)	101 (17.4)	1.55 ± 1.31
**Substance consumption in last 12 months**	159 (29.6)	82 (13.9)	54 (9.1)	124 (20.0)	172 (29.1)	3.11 ± 1.60

^a^ Data are presented as Mean ± SD or No. (%).

## 5. Discussion

The goal of this study was to assess relapse high risk situations in public and private substance abuse treatment centers in a southern province of Iran (Bushehr). A survey showed that 73% of them have used drugs in the past year. So, better monitoring and supervision should be done more effectively and should be accompanied with the participation of the families. In this regard, a valid test of drug control, monitoring the behavior of addicts and full compliance with the treatment protocols can also be useful.

It is worrisome that relapse rates of 60 to 100% are reported for the drug abusers. Basically, relapse prevention is a cognitive behavioral therapy approach, which requires expertise and special skills. Promoting coping skills can also be beneficial especially in high-risk situations. Identifying relapse high risk situations is an important strategy which can be attempted and provides the basis of their treatment. In this way, physicians can identify situations which are associated with a higher risk of recurrence through investigating the addict’s profile. Then, appropriate medical and psychological measures can be under taken to reduce relapse. Based on our findings, intrapersonal factors are somehow more important compared with interpersonal factors. These findings are consistent with those of the McKay et al. study (14). This suggests that patients’ interactions with the environment have a greater role in relapse. Intrapersonal factors include conflict with others e.g. when he/she had an argument with family or friends, pleasant times with others such as when person goes to a party and social pressures as when someone offered alcohol or drugs. So clinicians should pay more attention to these situations.

When we look carefully at Figure 1 that shows addicts’ profiles, we found that is similar to an undifferentiated flat profile in terms of the definition, because this facade is almost flat without any obvious ups and downs. It is also undifferentiated; because the severity of the problems is less than 60 percent. However, there is a difference of about twenty percent between the high risk situations with highest and lowest frequency, all of these situations contribute to relapse and it is necessary to address all of them. In this regard, the most critical situations are unpleasant emotions such as anger, frustration, boredom, sadness or anxiety that can lead the former addicts to resume drug abuse. In Hartwell’s study, physical discomfort is the most important relapse predisposing situation in opium addicts (11). It is recommended that psychologists apply the necessary interventions to control these emotions in partnership with families. Physical discomfort, including pain and illnesses are also triggers for relapse. In this regard, medical assistance and support should be provided. In contrary to the commonly held belief that addicts are seeking enjoyment and and happiness from drugs, according to our results pleasant emotional situations are not among the relapse related situations. 

Our findings showed that a significant percentage of addicts in treatment centers received just one type of psychological or clinical treatment. It is better to apply both types of treatment to increase the effectiveness and reduce relapse, as current substance abuse treatment protocols also endorse this notion. According to our findings, despite the existing documents indicating that psychological interventions are undertaken in treatment centers, it was not re-approved by the patients’ statements. Therefore, appropriate and adequate medical and psychological visits are encouraged.

In sum, addiction treatment is a combined process which can be achieved through addicts’ willingness to quit, family participation and clinical and psychological treatment. It seems necessary to monitor treatment process through diagnostic tests and therapeutic interventions. Identification of relapse-related high-risk situations and installing the appropriate medical and psychological measures to treat the addicts based on the extracted profile and reducing their relapse rate are effective strategies. In conclusion, promoting patients’ self-efficacy skills in order to cope with high-risk situations is recommended.

## References

[1] Dackis C, O'Brien C (2005). Neurobiology of addiction: treatment and public policy ramifications.. Nat Neurosci..

[2] Naderi N, Binazadeh M, Sefatian S, Asghar Peyvandi A (2009). Comprehensive text book of addiction: Dependence to different substances and their pharmacological and non- pharmacological treatment..

[3] Pringle JL, Edmondston LA, Holland CL, Kirisci L, Emptage NP, Balavage VK (2002). The role of wrap around services in retention and outcome in substance abuse treatment: Findings from the Wrap Around Services Impact Study.. Addict Disord Their Treat..

[4] Marlatt GA (1996). Taxonomy of high-risk situations for alcohol relapse: evolution and development of a cognitive-behavioral model.. Addiction..

[5] Marlatt GA, Gordon JR (1985). Relapse Prevention: Maintenance Strategies in the Treatment of Addiction Behavior..

[6] Larimer ME, Palmer RS, Marlatt GA (1999). Relapse prevention. An overview of Marlatt's cognitive-behavioral model.. Alcohol Res Health..

[7] Ibrahim F, Kumar N (2009). The influence of community on relapse addiction to drug use: evidence from Malaysia.. Eur J Soc Sci..

[8] Sadegiye AS, Azami A, Barak M, Amani F, Firuz S (2004). Reviewing the causes of recurred addiction in patients who introduced of Tehran welfare.. Ardabil Med Univ referred to centers J..

[9] El-Sheikh Sel G, Bashir TZ (2004). High-risk relapse situations and self-efficacy: comparison between alcoholics and heroin addicts.. Addict Behav..

[10] Kaplan H, Sadock B (1999). Kaplan Psychology..

[11] Hartwell KJ, Back SE, McRae-Clark AL, Shaftman SR, Brady KT (2012). Motives for using: a comparison of prescription opioid, marijuana and cocaine dependent individuals.. Addict Behav..

[12] Ghaemmohammadi MR (2004). The study of individual and social factors associated with relapse among addics outpatient drug rehabilitation and treatment center Abarkooh city..

[13] Annis H, Martin G (1985). Inventory of drug-taking situations.

[14] McKay JR, Murphy RT, McGuire J, Rivinus TR, Maisto SA (1992). Incarcerated adolescents' attributions for drug and alcohol use.. Addict Behav..

